# Different Neuroprotective Activities of Proanthocyanidin-Enriched Fractions of *Lotus* Species

**DOI:** 10.1021/acsomega.5c06714

**Published:** 2025-12-11

**Authors:** Maria Rachele Ceccarini, Nadia Mazzarella, Serena Visone, Pamela Santonicola, Antonella Camera, Federica Cieri, Federica La Rocca, Ilenia Matino, Giuseppina Zampi, Maria Cristina Valeri, Francesco Damiani, Francisco Jose Escaray, Oscar Adolfo Ruiz, Stefan Martens, Tommaso Beccari, Elia Di Schiavi, Francesco Paolocci

**Affiliations:** † Department of Pharmaceutical Science, 60251 University of Perugia, Perugia 06122, Italy; ‡ 509065 Institute of Biosciences and Bioresources, IBBR Naples Division CNR, Via P. Castellino 111, Napoli 80131, Italy; § Department of Biology, University of Naples “Federico II”, Napoli 80138, Italy; ∥ Department of environmental,biological and Pharmaceutical Sciences and Technologies, University of Campania “L. Vanvitelli, Caserta 81100, Italy; ⊥ 366183 Institute of Biosciences and Bioresources, IBBR Perugia Division CNR, Via Madonna Alta 130, Perugia 06128, Italy; # 62873 Chascomús Technological Institute (INTECH), School of Nanotechnology and Biotechnology (unsam-CONICET), Chascomús, Buenos Aires B7130, Argentina; ∇ 377369 Fondazione Edmund Mach, Centro Ricerca E Innovazione, Via E. Mach1, San Michele all’ Adige, Trentino 38098, Italy

## Abstract

Flavonoid-rich *Lotus* species are promising sustainable sources of bioactive phytochemicals due to their adaptability, high biomass production, and symbiosis with nitrogen-fixing *Rhizobium spp*. Among flavonoids, many beneficial effects for human health, ranging from antioxidant activities to the inhibition of carcinogenesis, are attributed to proanthocyanidins (PAs). This study compared the neuroprotective properties of leaf extracts from PA-rich *Lotus corniculatus* (*Lc*), PA-depleted *Lotus tenuis* (*Lt*), and *Lc* × *Lt* interspecific hybrid (*Lh2*) with intermediate PA levels. Acetone-soluble and -insoluble fractions from *Lc* and *Lh2* contained flavan-3-ols and PA oligomers, while *Lt* lacked these compounds. Neuroprotective assays in SH-SY5Y cells and *Caenorhabditis elegans* revealed that *Lc* and, although to a lesser extent, *Lh2* extracts enhanced cell viability and reduced motoneuron degeneration, whereas *Lt* extracts exhibited cytotoxicity and did not induce motoneuron viability rescue in *C. elegans*. Further analysis confirmed that pure flavan-3-ols, which represent the main components of the acetone-soluble fraction in *Lc* and *Lh2*, and cyanidin, which derives from the hydrolysis of their insoluble fractions, significantly promoted neuronal survival, while the flavonol quercetin showed no protective effects. These findings highlight the neuroprotective potential of PA-rich *Lotus spp*. and suggest their application as novel sources of health-promoting phytochemicals.

## Graphical abstract

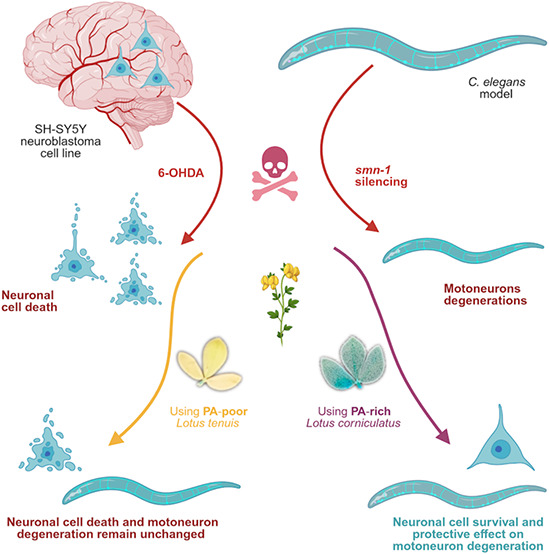

## 1 Introduction

The growing human population and the increasing human life expectancy are expected to cause a population of 10 billion in 2050 and 11.2 billion in 2100.^1^ On a global scale, the decrease of arable land worldwide, coupled with the plant cultivation constrains due to climate change, are other pressing issues that call for the exploitation of neglected or underutilized plant species to sustainably forefront the increasing consumer’s demand of both energy and phytochemicals.^2^ The last few decades have therefore witnessed increasing efforts to characterize through nutritional and medical evidence, different parts of plants and different plant species as potential source of phytonutrients.^3^

Due to their antioxidant properties and their marked effects in the prevention of various oxidative stress-associated pathologies, plant polyphenols have drawn increasing attention as phytonutrients.^4^ Among phenolic compounds, flavonoids are the most abundant polyphenols in our diet.^5^ They comprise flavones, flavonols, flavan-3-ols, flavanones, dihydroflavonols, isoflavones, anthocyanins, and condensed tannins, also known as proanthocyanidins (PAs). PAs are oligomers and polymers of the flavan-3-ols [e.g., catechin (C), epicatechin (EC)], hereinafter collectively referred to as catechins, linked through an interflavan carbon bond.^6^ Fruits like grapes, apples, pears, cherries, and green tea are some major sources of PAs and their monomers,^7^ which have been shown to protect against neurodegenerative diseases and the risk of cardiovascular disease^8,9^ and to have antibacterial activity against Gram-positive bacteria.^10^ Moreover, green tea catechins have been found to inhibit carcinogenesis of the skin, lung, esophagus, stomach, liver, small intestine, colon, bladder, prostate, and mammary glands in animal studies;^11^ epigallocatechin-3-gallate (EGCG) has been reported to have many potential targets against carcinogens;^12^ and EC in combination with exercise proved to improve memory function in mice.^13^ Recently it was also shown that condensed tannins from *Mitragyna speciosa* exert a potent virucidal activity against SARS-CoV-2.^14^

In foodstuffs, PAs are present as acetone extractable (also known as soluble) and nonextractable (also known as insoluble) fractions, the latter including highly polymerized molecules generally bound to other components of the food matrix, such as fibers. Most of the studies concerning PA metabolism have assumed that the PAs in foodstuffs correspond exclusively to the soluble fraction. However, recent studies have shown that a considerable proportion of PAs remains in the residue after acetone extraction and that this fraction might play a more significant functional role than the soluble fraction.^15^ The bioavailability of PA dimers and trimers has been the most investigated and once they reach the colon, they are widely transformed by the colonic microbiota into small phenolic acids,^16^ which are absorbed, transformed in the liver, and the resulting conjugates transferred to the bloodstream.^17^ Notwithstanding other studies have highlighted that polymeric PAs are also depolymerized into respective monomers, e.g., C and EC units from procyanidins, before cleavage into smaller species and further metabolism.^18^ Thus, it was argued that phenolics that are bioavailable after the ingestion of PA-rich foodstuffs must have come from nonextractable fraction.^19^

The genus *Lotus* comprises forage legumes grown worldwide, which can synthesize diverse classes of flavonoids, although to a different extent and with different organ-specificity.^20^ Due to their capacity to complex the protein of forage legumes, PAs play a critical role in controlling the fermentative processes in the rumen.^21^ Moreover, PAs possess anthelmintic properties.^20^ The commercial cultivars of *Lotus corniculatus* generally accumulate necessary levels of PAs in the range for preventing ruminal bloating; conversely, the levels of PAs in the herbage of *Lotus tenuis* are negligible.^22^ The interspecific hybrids between a wild accession of *L. corniculatus* with very high levels of PAs in the herbage and a PA-depleted cultivar of *L. tenuis*, largely grown in South America, have been produced. The molecular and metabolic analyses of the resulting hybrids have shown that PAs in *Lotus* herbage are a quantitatively inherited trait, with hybrids showing intermediate PA levels with respect to their parents and several regulatory genes implicated.^23,24^ Thus, we reasoned that the parents and their hybrids provided us the opportunity to test whether and to what extent the enriched PA fractions from PA-polymorphic but closely related *Lotus* accessions could exert any beneficial effects on human health, including neurodegenerative diseases. For the purpose of the present study, we used an *in vitro* model of Parkinson’s Disease (PD) and an *in vivo* model for Spinal Muscular Atrophy (SMA), in order to exploit the effect of *Lotus* PAs in different models and in different neurodegenerative conditions.

PD is the second most common neurodegenerative disorder after Alzheimer’s disease. The incidence of PD varies by region and ethnicity, but it is estimated that around 1–3% of people over the age of 60 are affected and this incidence is projected to double by 2040.^25^ The exact cause of PD is not fully understood, but a combination of genetic and environmental factors likely contributes to its development.^26^ While there is currently no cure for PD, treatment options are available to help manage symptoms and improve quality of life, including a role of diet in alleviating PD severity.^27^ The development of more valuable pharmacological strategies and novel personalized dietary integrations (e.g., polyphenol antioxidant to prevent dopaminergic neuron death) is highly demanded.

SMA is a rare genetic disorder that occurs mainly in childhood. The incidence of the disease is estimated to be approximately 1 in 6,000 to 1 in 10,000 live births each year.^28,29^ SMA is an autosomal recessive monogenic disease and mutations in the Survival Motor Neuron (*SMN1*) gene are the principal cause of the pathology, mainly characterized by the degeneration of motoneurons (MNs) of the anterior horns of the spinal cord resulting in muscle atrophy, paralysis, and, in the most severe forms, the death of the patients.^30^ Nowadays three therapeutic strategies have been approved by the US Food and Drug Administration (FDA) and the European Medicines Agency (EMA), and these approaches aim to increase SMN protein levels.^31^ Despite the great advances that have been made in SMA treatments that dramatically changed the disease trajectories and outcomes for severely affected infants, there are still several limitations; thus, further studies are still required to identify new potential drugs to be used in combination with the current treatments to increase their efficacy and reduce the side effects.

SH-SY5Y cell line, a subline of the SK-N-SH, is commonly used in neuroscience research, including PD.^32^ The SH-SY5Y cell line displays several genetic aberrations due to its cancerous origin, but most genes and pathways dysregulated in PD pathogenesis are intact.^33^ In this scenario, the SH-SY5Y cell line is widely used to investigate neuroinflammation, mitochondrial dysfunction and alpha-synuclein aggregation, which are hallmarks of PD development.^34^ Obviously, as an all *in vitro* model, it lacks the complexity of the human brain and all results should be interpreted cautiously and validated with a more complex *in vivo* model and, ultimately, in appropriate clinical trials.

An *in vivo* model largely used to test the impact of nutrients and natural extracts is the nematode *Caenorhabditis elegans*. In virtue of its short life cycle, body transparency and fast reproduction, *C. elegans* is also largely used as a genetically tractable *in vivo* model to study the molecular mechanisms of a variety of human diseases, affecting development and neuron function.^35,36^ Being an invertebrate, *C. elegans* fulfills to the principles of replacement, reduction, and refinement (3Rs) of experiments with mammals,^37^ thus it is also increasingly used in toxicology studies.^38,39^ Moreover, thanks to the high conservation of genes and pathways with humans, it is employed for the identification of new synthetic and natural compounds.^40–42^ Interestingly PAs have been tested multiple times on *C. elegans*,^43,44^ strongly supporting its use to uncover possible new applications for these natural compounds. In the present study, we used a *C. elegans* model for Spinal Muscular Atrophy (SMA), in which *smn-1,* the ortholog of *SMN1*, has been silenced via RNAi specifically in 19 motoneurons.^45^ These animals display an age-dependent degeneration of motoneurons (MNs) detected as altered locomotion, the early disappearance of neurons expressing the green fluorescent protein, and finally the apoptotic death of targeted neurons. Unlike other *C. elegans* genetic models of SMA,^46,47^ this approach removes the hindrances due to the pleiotropic phenotypes and, at the same time, allows the exploitation of the advantages of the nematode, and the rapid identification of the factors preventing the death of the MNs *in vivo*.^48,49^ This *C. elegans* model turned out to be effectual in identifying the genetic pathways involved^50,51^ and in discriminating the efficacy of green from gold kiwi extracts in decreasing the extent of neuronal death, which worked better than valproic acid, a well-established drug successfully used in cell cultures, mouse, and *C. elegans* SMA models.^52,53^

Thus, to test the hypothesis that *Lotus* PA might represent a novel and sustainable source of phytochemicals here we employed both the acetone-soluble (mainly containing free flavan-3-ols and oligomers) and the butanol-hydrolyzed acetone-insoluble fraction of leaves from PA polymorphic, but phylogenetically related *Lotus spp*., on the two models for the study of neurodegenerative processes.

Overall, our results show that PA-rich *Lotus* extracts yield neuroprotective effects on SH-SY5Y cells and preserve *C. elegans* motoneurons from death. Additionally, the pure compounds C, EC, and Cya, which are the main constituents of the acetone-soluble and insoluble fractions of the PA-rich *Lotus spp*., respectively, not only exert a neuroprotective effect toward the models cited above but also when SH-SY5Y cells are challenged with the neurotoxic drug 6-OHDA. Thus, the present study lays the ground to the exploitation of *Lotus* PAs as a novel and sustainable source of neurodegenerative-protectant nutraceutical compounds.

## 2 Methods

### 2.1 Plant Material and Reagents

Parental *L. corniculatus* (*Lc*) and *L. tenuis* (*Lt*) plants and the *L. corniculatus* × *L. tenuis* *Lh2* hybrid employed in this study have been previously characterized.^22–24^ Plants were grown in a growth chamber with a photoperiod of 16 h of white light and a photosynthetic photon flux density (PPFD) of 450 μmol m^–2^ s^–1^ at 24 °C, followed by 8 h in the dark at 20 °C and relative humidity ranging from 55% to 65%. The plant material above was propagated by cuttings and then transferred to a glasshouse under outdoor environmental conditions. Aliquots of about 0.5 g of fresh leaf material were collected from a pool of plants for each line and then immediately frozen in liquid nitrogen. PAs isolation from at least three independent pools per line and experiment was performed as described by Li and colleagues (1996) with some modifications.^54^ Leaves were ground in a mortar and extracted in a 15 mL Falcon tube with 1.5 mL of 70% (v/v) acetone aqueous solution containing 0.1% (w/v) ascorbic acid at 4 °C for 120 min using a Rotamix (Rotamix RK, Hero, Canada). The extraction was then repeated three times (2 × 120 min and one overnight), and the supernatant of the extracts was collected by centrifugation (20 min at 15,000 rpm at 4 °C in a Beckman centrifuge). The supernatants of each genotype were pooled, adjusted to 4 mL with 70% (v/v) acetone, extracted with 3 mL of diethyl ether, and then the lower fraction was collected for subsequent analyses.^54^ The pellets resulting from the extraction of soluble PAs were vacuum-dried, mixed well with 200 μL of 1% (w/v) SDS and 1.2 mL of a butanol-HCl (19:1, v/v) reagent, and depolymerized oxidatively for 90 min at 95 °C to yield anthocyanidins as cleavage products. These cleavage products were collected by centrifugation and quantified spectrophotometrically along with the soluble fractions as in Li and colleagues (1996) using catechin (C) and cyanidin (Cya) as standards for acetone-soluble and acetone-insoluble fractions, respectively.^54^ Both of these fractions were then aliquoted in 2 mL Eppendorf tubes, dried using a Thermo Scientific Savant SpeedVac vacuum concentrator, and stored at −20 °C before their use when they were resuspended in DMSO for *in vivo* and *in vitro* analyses.

### 2.2 Plant Extracts Characterization

1 mL aliquot of acetone-insoluble and soluble fractions from three replicates per each *Lotus spp*. from a single experiment underwent a targeted UPLC-DAD analysis for the characterization of their anthocyanin content using Cya, Pelargonidin, Paeonidin, Delphinidin, and Malvidin as authentic standards for cochromatography according to the method described in Rafique et al. (2016).^55^ Briefly, anthocyanin analysis was accomplished under gradient conditions on a Nucleodur C18ec column (250/4 Macherey-Nagel; Dueren, Germany) with solvent A 1% phosphoric acid in water and solvent B 1% phosphoric acid in acetonitrile. The gradient starts with 100% A to 50% A in 25 min, a plateau of 3 min, up to 100% A in 7 min, and a final plateau of 5 min with a flow rate of 1 mL min^–1^ and monitored at 280 and 520 nm. Flavonols, flavan-3-ol monomers, and PAs were determined on the acetone-soluble fractions by a validated targeted Ultra-Performance Liquid Chromatography-tandem mass spectrometer (UPLC-MS/MS) on a Waters Acquity system (Milford, MA, USA) using a Waters Acquity HSS T3 column (1.8 μm, 100 × 2.1 mm^2^, set at 40 °C) and the separation conditions as previously described.^56^ Data processing was performed using the MassLynx TargetLynx Application Manager (Waters). All authentic standards used in the two chromatographic methods, i.e., C, EC, quercetin (Que), other flavonols, procyanidin derivatives, and the anthocyanins Cya, delphinidin, pelargonidin, and paeonidin, were obtained from TransMIT PlantMetaChem (Giessen, Germany).

### 2.3 *In Vitro* MTT Assay

The human neuroblastoma cell line SH-SY5Y, obtained from the American Type Culture Collection (ATCC, Manassas, VA, USA), was used to investigate C, EC, Que, and Cya molecules and the extracts from *Lotus spp*. for cytotoxicity. SH-SY5Y cells were tested for mycoplasma contamination and then cultured according to standard procedures in Roswell Park Memorial Institute 1640 medium (RPMI-1640). The medium was supplemented with 10% heat-inactivated Fetal Bovine Serum (FBS), 2 mM of l-glutamine and antibiotics (100 U/mL penicillin, 100 μg/mL streptomycin; Gibco, Invitrogen, Carlsbad, CA, USA). The cell line was cultured at 37.0 °C in a 5% CO_2_ atmosphere, and the medium was replaced every 3 days.

SH-SY5Y cells were seeded at the density of 5 × 10^3^ cells/well into 96-well flat-bottom culture plates in a final volume of 200 μL. After 24 h the cells were treated with 200 μL of all samples and maintained for 24 h.^57^ MTT was used in a final concentration of 0.5 μg/μL for 3 h and at the end the supernatant was trashed away, and the cells were lysed with 100 μL of DMSO as previously described.^34,58^ The absorbance (OD) values were measured spectrophotometrically at 540 nm using a spectrophotometer reader (Jenway 6715 UV/vis, Bibby Scientific Ltd., Dunmow, UK) and cell viability was expressed as a percentage relative to that of the control cells.^59^ Each experiment was performed in triplicate, and the results were expressed as a percentage relative to those of the control cells. In order to exclude false positives, 1% and 2% DMSO were used as positive controls.^60^ 6-OHDA, dissolved in water with 1% DMSO, according with the user’s manual, is commonly used to selectively kill dopaminergic and noradrenergic neurons. 6-OHDA was investigated in a dose–response curve (25, 50, 100, 150 μM) and for all further stimulations the 50 μM concentration was used as previously reported.^34^ C, EC, and Cya were resuspended in 1% DMSO and tested at 50, 100, 150, and 200 μM, whereas Que was resuspended in 1% DMSO and tested at 25, 50, 100, and 150 μM. Acetone-soluble and butanolic fractions of *Lt*, *Lh2*, and *Lc* were resuspended in 200 and 300 μL of DMSO, respectively, then diluted to 1% (Ac) and 2% DMSO (Bu) with the cell medium. The neurotoxic synthetic organic compound 2,4,5-trihydroxyphenethylamine (6-OHDA) was obtained from Sigma-Aldrich (Milan, Italy).

### 2.4 *C. elegans* Strains and Nematode Maintenance

The wild-type *C. elegans* strain used in this work was N2, variety Bristol; the transgenic *C. elegans* strains used in this work were: NA1330 *gbIs4* [p*unc-25:smn-1*(RNAi sas); p*chs-2:GFP*] III, which presents *smn-1* knock down in D-type GABAergic motoneurons; and NA1355 *gbIs4* III; *oxIs12* [p*unc-47:GFP*; *lin-15*(+)] X, which in addition presents green fluorescent protein (GFP) expression in D-type GABAergic motoneurons.^45^ Nematodes were grown under standard conditions, at 20 °C ± 1.5 °C on NGM (Nematode Growth Medium) plates seeded with bacteria, *Escherichia coli* strain OP50.^61^

### 2.5 Influence of Extracts and Individual Components on *C. elegans* Neurodegeneration

Lyophilized *Lotus spp*. acetone-soluble and butanolic fractions were dissolved in M9 buffer, filtered using 1.2 μm membrane filters (Millipore), and then sterilized by filtration with 0.22 μm membrane filters (Millipore). All filtered extracts were then added to plates containing solidified agar with NGM to reach the final dilution.^52^ Extracts were allowed to adsorb for 1 day, and then heat-killed OP50 bacteria were added. Individual molecules were dissolved in DMSO and similarly added to plates at the final concentration described. DMSO 1% or water was added on separate plates as negative controls (mock treatments) and tested every time. C, EC, and Que concentrations were chosen based on literature.^62^ At 800 μM all compounds were toxic for embryogenesis, and no eggs hatched, so we used concentrations up to 400 μM, which were compatible with animal survival and fertility. Ascorbic acid and valproic acid were diluted in water. The evaluation of *C. elegans* neurodegeneration was performed as already reported^45^ by using control animals treated with mock on the same day and analyzing two different phenotypes: (i) an age-dependent degeneration of motoneurons (MNs) detected as the disappearance of neurons expressing the green fluorescent protein from transgene *oxIs12* (early event); (ii) the apoptotic death of targeted neurons in the absence of the transgene *oxIs12* (late event). The latter phenotype is detectable as the accumulation of GFP in dying motor neurons, an unexpected and novel phenotype whose specific dependence on the knockdown of *smn-1* and correlation with apoptosis has been largely demonstrated.^45^ The first-generation progeny was evaluated for neuron death at the stage of a young adult. Each experimental condition was run blindly in duplicate or in triplicate and repeated by at least two different independent observers. The neuroprotective drugs valproic and ascorbic acid used for *in vivo* tests were obtained from Sigma-Aldrich (Milan, Italy).

### 2.6 Microscopy Analysis

Animals were immobilized in 0.01% tetramisole hydrochloride (Sigma-Aldrich) on 4% agar pads and visualized using a Zeiss Axioskop equipped with epifluorescence and DIC Nomarski optics. To discriminate dying motoneuron fluorescence (late event) from endogenous autofluorescence, a Zeiss filter set 09 was used. This setting allowed the observation of intestinal cell autofluorescence in yellow and apoptotic-fluorescence-positive dying cells in green. The early degenerative phenotype was scored by counting the number of visible D-type motoneurons expressing GFP per animal using a motoneuron-specific promoter (*punc-47:GFP*). The number of animals analyzed each time (*n*) was added to graphs.

### 2.7 Statistical Analysis

GraphPad Prism software was used for the statistical analysis. The statistical significance was determined using ONE-way ANOVA followed by the Bonferroni posttest or Kruskal–Wallis test, *z* statistic, or Unpaired *t*-tests, comparing each sample against the control. The standard error of the mean was used to estimate variation within a single population in different experiments. The standard deviation was used to estimate variation within a population in a single experiment.

## 3 Results

### 3.1 Metabolic Characterization of *Lotus* Extracts

The content of acetone-soluble and -insoluble fractions in the leaves of the *Lotus* material grown in the glasshouse remained quite stable over the entire season of leaf collection (June–July 2016). The acetone-soluble PA fraction measured through spectrophotometric analysis and expressed as mg of C equivalents per g of fresh weight turned out to be higher in *Lc* than in *Lt* and intermediate in *Lh2*, as expected^22,23^ (Table 1). Likewise, the quantification of the acetone-insoluble fraction, expressed as Cya equivalent per g of fresh weight (FW), not only showed that these fractions were stable within each species across the sampling season but also confirmed the differences among the species. As such, Cya levels from *Lc* fractions were higher than those in *Lt* and intermediate in *Lh2* (Table 1).

**Table 1:** Acetone-Soluble and Insoluble Fractions of *Lotus spp* ^a^

Samples	Acetone-soluble fraction (mg/g FW)^b^	Acetone-insoluble fraction (mg/g FW)^b^
*Lc*	2.256 ± 0.45	2.086 ± 0.33
*Lt*	0.002 ± 0.00	0.061 ± 0.02
*Lh2*	0.186 ± 0.11	1.732 ± 0.30

a Assessed by spectrophotometric analysis .

b Average levels ± SE from two leaf harvestings with 3 biological replicates per harvesting.

To gain a glimpse on the composition of these extracts, UPLC-DAD (anthocyanidins) and targeted LC-MS/MS (PAs, their monomers, and flavonols) analyses were run. Anthocyanidins were not detectable in any acetone-soluble fraction. As illustrated in Supplementary Figure S1, the UPLC-DAD analysis of acetone-insoluble fractions after butanol-HCl hydrolysis confirmed the absence of detectable anthocyanidin peaks in the acetone-insoluble fraction of *Lt* (A), the presence of Cya and, although below the level of quantification, of delphinidin in that of *Lh2* (B) and of both Cya and delphinidin in that of *Lc* (C) (Table 2 and Supplementary Figure S1). Two unknown anthocyanidin peaks, which might be oligomers or polymers of anthocyanins,^14^ were also detected in both *Lh2* and *Lc* fractions. Due to their small amounts, the quantification of these peaks was precluded (Supplementary Figure S1).

**Table 2:** Targeted LC-MS/MS and UPLC-DAD Quantification of PA Soluble and Insoluble Fractionof *Lotus* spp

	PA soluble fractions (mg/100 FW)^a^									PA insoluble fractions (mg/100 dry weight)^b^	
Samples	Catechin	Epicatechin	Procyanidin B1	Procyanidin B2 + B4	Kaempferol-3-Glc	Quercetin-3-Rha	Quercetin-3-GlcAra	Quercetin-3,4-diglucoside	Isorhamnetin-3-Glc	Cyanidin	Delphinidin
***Lt***					0.111 ± 0.01	0.081 ± 0.01	0.087 ± 0.02	0.019 ± 0.01	0.016 ± 0.004		
***Lh2***	0.125 ± 0.03	0.350 ± 0.03	0.143 ± 0.09	0.252 ± 0.05	0.681 ± 0.10	0.067 ± 0.02	0.036 ± 0.01	0.092 ± 0.03	0.383 ± 0.03	126 ± 52.85	
***Lc***	0.046 ± 0.03	0.444 ± 0.21	0.101 ± 0.09	0.417 ± 0.22	0.335 ± 0.11	0.490 ± 0.13		0.277 ± 0.10	0.990 ± 0.27	235.5 ± 24.75	231 ± 39.60

a Data from LC-MS/MS analysis.

b Data from UPLC-DAD analysis of butanol-HCl hydrolyzed, freeze-dried pellets after the extraction of soluble PAs.

Targeted LC-MS/MS analysis confirmed the absence of detectable levels of flavan-3-ols or PA dimers in the acetone-soluble fraction of *Lt*, the presence of EC, procyanidin B1, B2, and B4 from the acetone-soluble fraction of *Lh2*, and that of both C and EC as flavan-3-ols, along with procyanidin B1, B2, and B4 from the acetone-soluble fraction of *Lc* (Table 2). Procyanidin B1 consists of a molecule of EC and C joined to each other by a bond between positions 4 and 8′ in a β-configuration, procyanidin B2 consists of two molecules of EC joined by a bond as above, and procyanidin B4 is a C-(4α→8)-EC dimer. Notably, procyanidin B2 and B4 were more abundant in *Lc* than in *Lh2*. Since by targeted analyses only flavan-3-ols, PAs, flavonols, and cyanidins recognized by the authentic standards included in the respective database could be identified, other derivatives, especially oligo- and polymeric PAs, could not be detected even if present in high concentration in the extracts. Therefore, we relied on spectrophotometric data for the estimation of C and Cya equivalents for subsequent experiments.

### 3.2 Pure Flavan-3-ols Do Not Affect the Viability of Human SH-SY5Y Cell Line but Reduce the Number of Dying Neurons *In Vitro* and in a *C. elegans* Model of SMA

Because the acetone-soluble fraction of *Lotus spp*. mainly contained and differed each from the other for the content of free flavan-3-ols, C and EC, and PA oligomers, we verified at first any potential negative effects of free flavan-3-ols on human cell lines. Likewise, as through hydrolysis in butanol-HCl the acetone-insoluble PA fraction released anthocyanins, Cya was also employed. Thus, increasing doses of commercially available C and EC, ranging from 2.90 μg for C and EC at 50 μM, to 11.61 μg for C and EC at 200 μM, and Cya, from 3.22 μg at 50 μM to 12.88 μg at 200 μM, were tested on the SH-SY5Y cell line, along with the flavonol Que, as a control. In each of these experiments, this cell line was challenged with 1% and 2% DMSO as control (Figure 1a). Not only C, EC, and Cya turned out to be not toxic, but at 200 μM they all increased cell viability with respect to DMSO 1% (Figure 1b, c, e). Conversely, Que did not significantly affect cell viability at any of the concentrations tested, which ranged from 1.51 μg (50 μM) to 9.06 μg (150 μM)­(Figure 1d).

**Figure 1:**
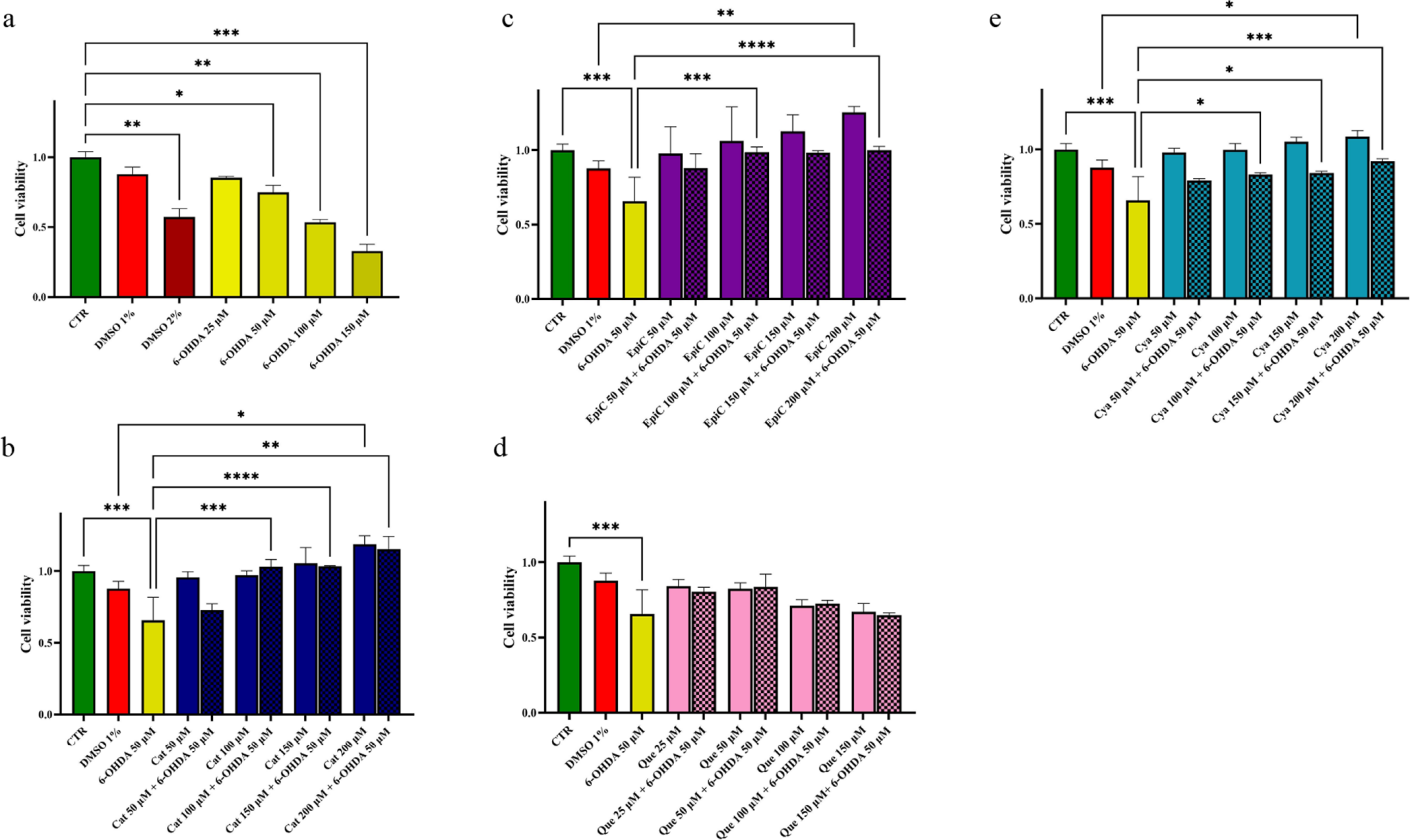
Pure flavonoid compounds differently affect the viability of SH-SY5Y cells grown under control and 6-OHDA-supplemented media. MTT assay on SH-SY5Y cell line after 24 h of treatment. Negative control (in green), and positive control (in red) were always reported. a) 6-OHDA from 25 to 150 μM (in yellow), b) C from 50 to 200 μM alone (in blue) and in combination with 50 μM 6-OHDA (squares in blue), c) EC from 50 to 200 μM alone (in violet) and in combination with 50 μM 6-OHDA (squares in violet), d) Que from 25 to 150 μM alone (in pink) and in combination with 50 μM 6-OHDA (squares in pink), and e) Cya from 50 to 200 μM alone (in light blue) and in combination with 50 μM 6-OHDA (squares in light blue). Dunnett’s T3 multiple comparisons test was done. The significance thresholds were set as follows: *p < 0.05, **p < 0.01, ***p < 0.001, and ****p < 0.0001.

To assess whether C, EC, Que, and Cya could interfere with the neurotoxic activity of 6-OHDA on the SH-SY5Y cell line, we first challenged these cells with 25, 50, 100, and 150 μM of 6-OHDA (Figure 1a). After 24 h of incubation, cell viability under 50 μM 6-OHDA treatment and, to a far more extent, 100 and 150 μM 6-OHDA significantly decreased to values even lower than those achieved with 1% DMSO (Figure 1a). Therefore, in the following experiments, the effects of increasing doses of the pure compounds C, EC, Que, and Cya were tested on SH-SY5Y challenged with 50 μM 6-OHDA (Figure 1b, c, d, e). A 100 μM concentration of C, EC, and Cya was sufficient to fully contrast the negative effect of 50 μM of 6-OHDA (Figure 1b, c, e). This effect was also obtained with 150 and 200 μM C and Cya and 200 μM EC. Conversely, the addition of Que did not induce a significant increase of viability in cells treated with 50 μM 6-OHDA, at any of the concentrations tested (Figure 1d).

To test the neuroprotective effects exerted by flavan-3-ols in a whole organism and verify whether their action is restricted to 6-OHDA-induced degeneration or has a broader effect, we turned to *C. elegans*. We took advantage of the *C. elegans* SMA model we developed^45^ and tested whether chronic treatment with flavan-3-ols could prevent the apoptotic death of motoneurons (MNs). *smn-1* silencing in MNs causes the death of neurons, detectable as the late accumulation of apoptosis-related fluorescence in dying MNs, whose nature has been confirmed using cell-death markers and genetic mutants and is never observed in negative controls.^45^ In line with the effects observed in the human cell line, C, EC, and Cya displayed a different activity with respect to the flavonol Que when assayed on the *C. elegans* SMA model (Figure 2a, b). 200 μM C, EC, and Cya significantly decreased the number of dying motoneurons of more than 25%, whereas similar levels of Que did not (Figure 2a). The increase of both C and EC at 400 μM decreased even further the number of dying neurons, whereas the inefficacy of Que was confirmed at 400 μM, a dosage at which Cya was also not efficient (Figure 2b). A similar neuroprotective role was obtained with ascorbic acid, a well-known antioxidant molecule that was able to rescue apoptotic death with a dose–response effect (Figure 2c).

**Figure 2:**
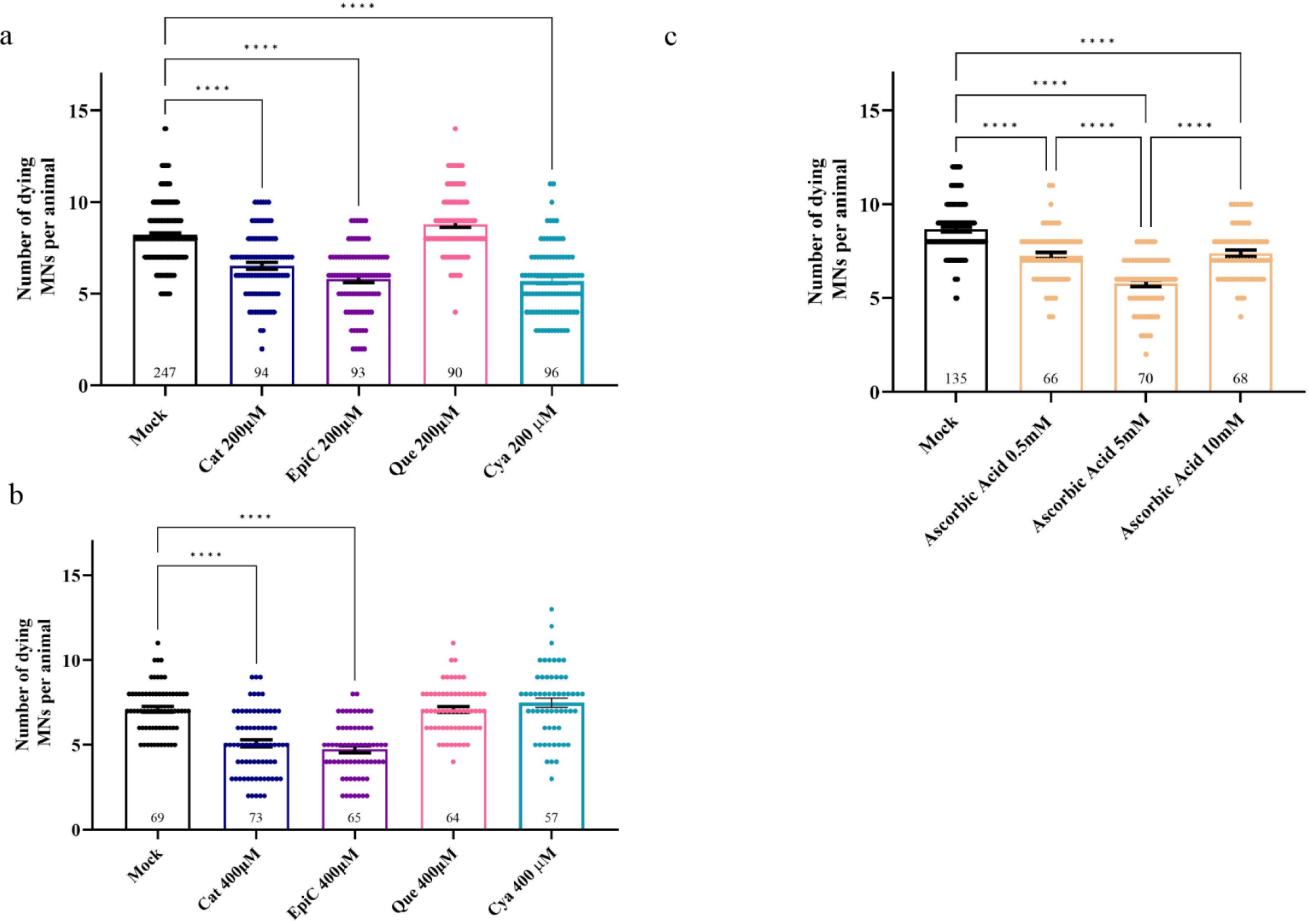
Catechins significantly reduced the number of dying motoneurons in a *C. elegans* SMA model. a) *smn-1­(MNs RNAi)* animals present 8.2 dying MNs in mock that are reduced when C, EC, and Cya are added at 200 μM, thus causing a rescue of the phenotype. Mock is 1% DMSO. b) C and EC rescued the number of dying motoneurons also at 400 μM. Mock is 1% DMSO. c) Ascorbic acid rescued MN death at 0.5, 5, and 10 mM. Mock is water. In all graphs the scatter plot, the means of dying neurons per animal, and the SEM are shown; *n* is the number of animals observed. Asterisks indicate a value significantly different (****p < 0.0001) from animals treated with solvent alone (mock), as calculated with One-Way ANOVA, Kruskal–Wallis nonparametric test, Dunn’s multiple comparison.

### 3.3 Extracts from Different *Lotus spp*. Played a Contrasting Action on Viability of Human Cell Lines

To test for any potential cytotoxic activity of both acetone-soluble and insoluble fractions from the three *Lotus* genotypes under investigation, increasing dosages of these fractions were added to the medium for growing the SH-SY5Y cell line and *C. elegans*. To this end, scalar volumes (25, 50, 100, and 150 μL) of acetone-soluble fractions were employed for the assays with the SH-SY5Y cell line. According to spectrophotometric analysis, these volumes correspond to 0.06, 0.14, 0.27, and 0.41 μg of C equivalent for *Lh2* and to 0.37, 0.75, 1.50, and 2.25 μg of C equivalent for *Lc*, respectively. Likewise, scalar volumes of acetone-insoluble fractions (25, 50, 100, and 150 μL) corresponding to Cya equivalents ranging from 0.08 μg (in 25 μL) to 0.49 μg (in 150 μL) for *Lh2*, and from 0.25 μg (in 25 μL) to 1.50 μg (in 150 μL) of *Lc* were also employed. No or subtle levels of C equivalents and Cya equivalents were present in the acetone-soluble and acetone-insoluble fractions of *Lt,* respectively.

While the acetone-soluble extracts of *Lt* did not affect cell viability at lower concentrations, they did so at higher concentrations, and the more extract was added, the greater the cell mortality was compared to that of the 1% DMSO treatment used as a control (Figure 3a). The decline in cell viability observed when increasing levels of acetone-soluble *Lt* extracts could not be accounted to the increasing levels of DMSO in the medium (up to 1.5% in samples with 150 μL of extracts), since such an effect was not recorded when similar amounts of *Lh2* and *Lc* extracts were added (Figure 3b, c). The addition of increasing doses of C (up to 30 μg) partially rescued the cell viability impairment caused by *Lt* extracts (Supplementary Figure S2). The acetone-soluble fractions of *Lh2* and *Lc* were not toxic at any dosage (Figure 3b, c). The addition of 50 μL of acetone-soluble fractions from either *Lh2* or *Lc* was not sufficient to increase the viability of cells treated with 150 μL of acetone-soluble fractions from *Lt* (Supplementary Figure S3). This is likely due to the low concentrations of C in *Lh2* and *Lc* fractions. As regards the acetone-insoluble fractions, those from *Lt* resulted to be cytotoxic at the highest doses (100 and 150 μL) (Figure 3d), whereas an opposite effect was detected for *Lh2* and *Lc*, as 150 μL of their fractions increased cell viability (Figure 3e, f). 30 μg portion of C rescued cell viability in the SH-SY5Y cell line treated with the insoluble fraction of *Lt* (Supplementary Figure S2). Likewise, adding 50 μL of acetone-insoluble fractions from either *Lh2* or *Lc* to that from *Lt* (150 μL) increased cell viability (Supplementary Figure S3). Thus, the cytotoxic effect of acetone-soluble and -insoluble *Lt* fractions can be counteracted by C, while only acetone-insoluble *Lt* fraction-mediated negative effects can be rescued by acetone-insoluble fractions obtained from *Lh2* and *Lc*.

**Figure 3:**
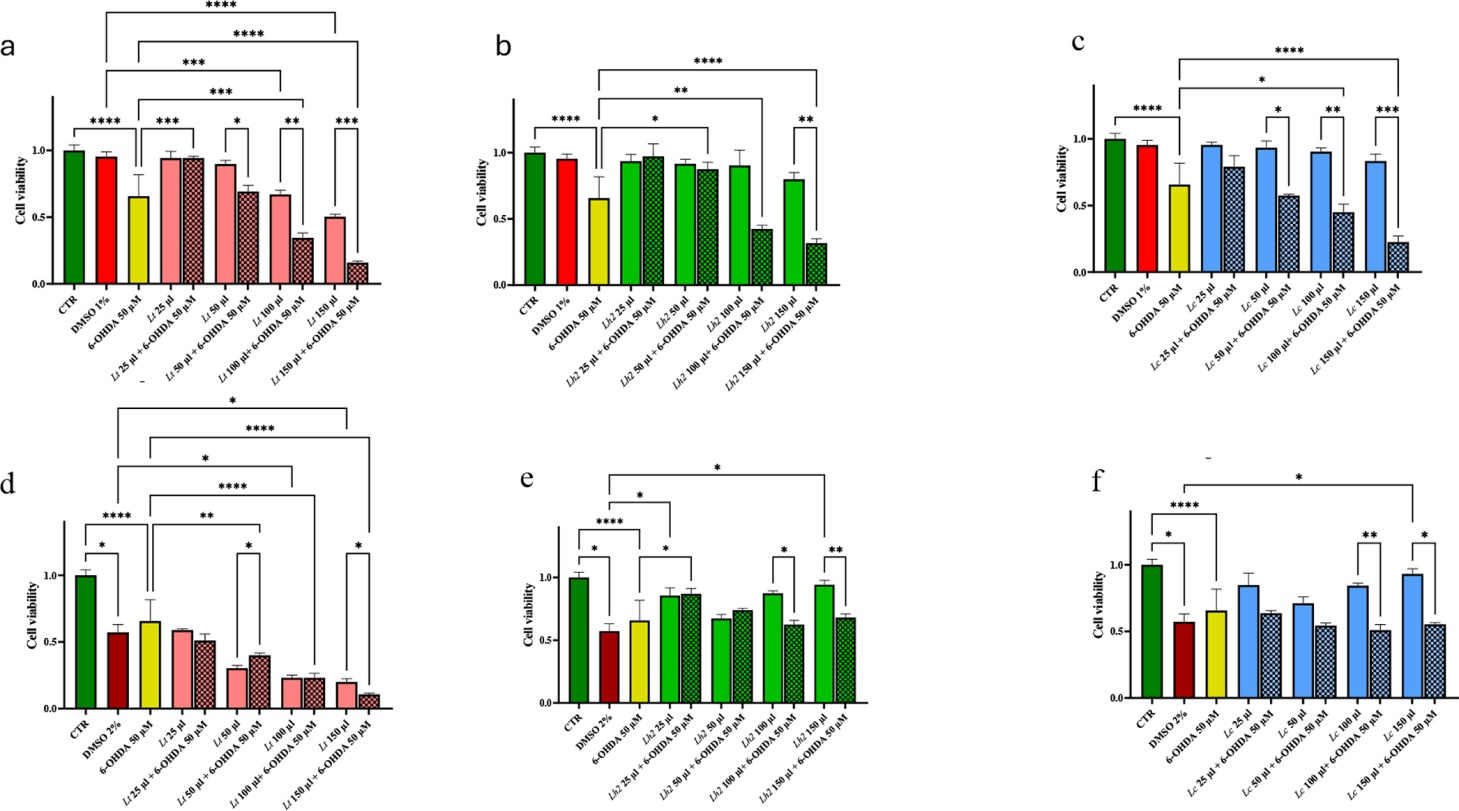
Effects of different dosages of acetone-soluble and acetone-insoluble extracts from *Lotus spp*. on the viability of SH-SY5Y cells grown under control and 6-OHDA-supplemented media. MTT assay on SH-SY5Y cell line after 24 h of treatment. Negative control (in green), positive control (in red), and 50 μM 6-OHDA-treated control (in yellow) were always reported. a) Acetone-soluble extracts from *Lt* alone (in violet) and in combination with 50 μM 6-OHDA (squares in violet), b) Acetone-soluble extracts from *Lh2* alone (in green) and in combination with 50 μM 6-OHDA (squares in green); c) Acetone-soluble extracts from *Lc* (in blue) and in combination with 50 μM 6-OHDA (squares in blue); d) Acetone-insoluble extracts from *Lt* (in violet) and in combination with 50 μM 6-OHDA (squares in violet); e) Acetone-insoluble extracts from *Lh2* (in green) and in combination with 50 μM 6-OHDA (squares in green); f) Acetone-insoluble extracts from *Lc* (in blue) and in combination with 50 μM 6-OHDA (squares in blue); The significance thresholds using Dunnett’s T3 multiple comparisons test were set as follows: *p < 0.05, **p < 0.01, ***p < 0.001, and ****p < 0.0001.

### 3.4 Dosage and Genotype-Dependent Effects of Acetone-Soluble and Insoluble Fractions on the Viability of SH-SY5Y Cells Challenged with 6-OHDA

To check whether the *Lotus* extracts counterbalance the negative effects of 6-OHDA on cell viability, increasing doses of both acetone-soluble and insoluble fractions from the three genotypes under investigation were tested on SH-SY5Y cells challenged with 50 μM 6-OHDA.^34^ As regards the acetone-soluble fractions, only the smallest amount of the extracts from each genotype was protective and/or not harmful toward the cells (Figure 3a, b, c). Cell viability was slightly higher with 25 μL of acetone-soluble extracts from *Lt* and *Lh2*, but it dropped to levels significantly lower than those of the control treatment when 100 or 150 μL of these extracts were added, regardless of the genotypes (Figure 3a, b, c). The result was different when the acetone-insoluble fractions were employed (Figure 3d, e, f). This fraction from *Lt* decreased cell viability with the increase of its amount in the medium, and this decrement was significant in the range of 50 to 150 μL. The same did not occur with the extracts from the other two *Lotus* genotypes. Once again, the decrease in cell viability observed with increasing doses of *Lt* fractions could not be accounted to the sole effects of DMSO. With the only exception for 25 μL of *Lh2*, a dosage that increased cell viability, the addition of the different amounts of butanolic extracts from the acetone-insoluble fractions from either *Lh2* or *Lc* did not significantly affect cell viability. However, by comparing the viability of the cells treated with 100 and 150 μL of these extracts from *Lh2* or *Lc* with that of the cells treated with the same amount of extracts in the presence of 6-OHDA, it can be concluded that these amounts of *Lh2* and *Lc* acetone-soluble extracts did not preserve the cells from the damage induced by 6-OHDA.

### 3.5 Extracts from *Lc* Enriched in Tannins Have a Neuroprotective Effect in the *C. elegans* Model for SMA

Transgenic *C. elegans* animals with *smn-1* silencing in MNs grown on the acetone-soluble extracts from the three *Lotus spp*. displayed a significant decrease in neuronal death only when those from *Lc* were used (Figure 4a). Conversely, the acetone-insoluble fractions of both *Lh2* and *Lc* showed a significant decrease in neuronal death as demonstrated by a reduction in the average number of dying MNs from 7.7, in the mock-treated animals, to 5.6 and 7 in the nematodes treated with the *Lc* and *Lh2* extracts, respectively (Figure 4b). The rescue of apoptotic death was observed only when a concentration of 1% (w/v) was used and not at higher or lower concentrations (not shown). Thus, both acetone-soluble and insoluble fractions of *Lc* were able to partially prevent the apoptotic death of MNs observed in a *C. elegans* SMA model.

**Figure 4:**
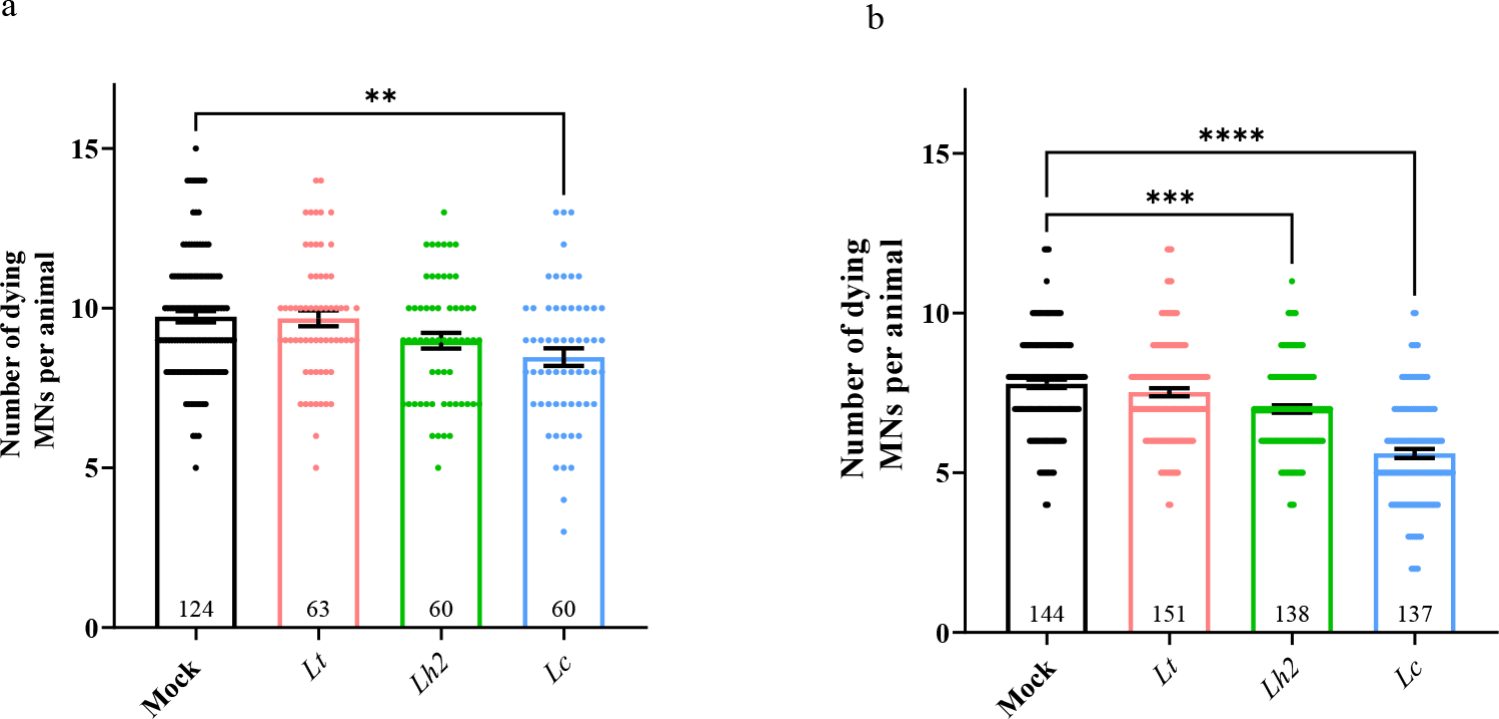
Total extracts from *Lc* significantly reduced the number of dying motoneurons in a *C. elegans* SMA model. a) *smn-1­(MNs RNAi)* animals present 9.7 dying MNs in mock that are reduced when the acetone-soluble fraction of *Lc* at 1% w/v concentration is added, thus rescuing the neuronal death. No effect is visible with other *Lotus* genetic backgrounds. b) Two independent preparations of *Lc* and *Lh2* extracts but not *Lt*, when hydrolyzed with butanol, rescue the *smn-1* silencing-induced death by reducing the number of dying motoneurons per animal from 7.7, in the mock-treated animals, to 5.6 and 7, respectively. In all graphs the scatter plot, the means of dying neurons per animal and the SEM are shown; mock is 1% DMSO; *n* is the number of animals observed. Asterisks indicate a value significantly different (**p = 0.0071; ***p = 0.0002; ****p < 0.0001) from animals treated with solvent alone (mock), as calculated with One-Way ANOVA, Kruskal–Wallis nonparametric test, Dunn’s multiple comparison.

### 3.6 Catechins Have a Late Antiapoptotic Effect in the *C. elegans* Model for SMA

C and EC were able to decrease the death of motoneurons in the *C. elegans* model, likewise the acetone-soluble fraction of *Lc*, which contains higher C equivalents than those from *Lt* and *Lh2* (Figures 2 and 4). To establish whether C was able to prevent neurodegeneration in the early stages, we turned to a more precocious phenotype observed in another SMA model, in which along with the knockdown of *smn-1* we also have GFP expression in viable MNs. In these double-transgenic animal’s early signs of degeneration are detectable as the disappearance of GFP in motoneurons and we investigated the morphology and viability of GFP-expressing MNs after exposure to catechins. By microscopy analysis we found no improvement in GFP-expressing MNs in the presence of C compared to the animals grown on the mock (Figure 5a), while valproic acid did rescue this early sign of neurodegeneration, as expected^45^ (Figure 5b). Interestingly, no effect was obtained with ascorbic acid, a well-known antioxidant molecule, which was able to rescue only apoptotic death (Figure 2b), but not the number of GFP-expressing MNs (Figure 5b). These results strongly suggest that C and EC present in the *Lc* acetone-soluble fraction are responsible for the rescue of apoptotic death that occurs at late stages after activation of the cell death pathway genes.

**Figure 5:**
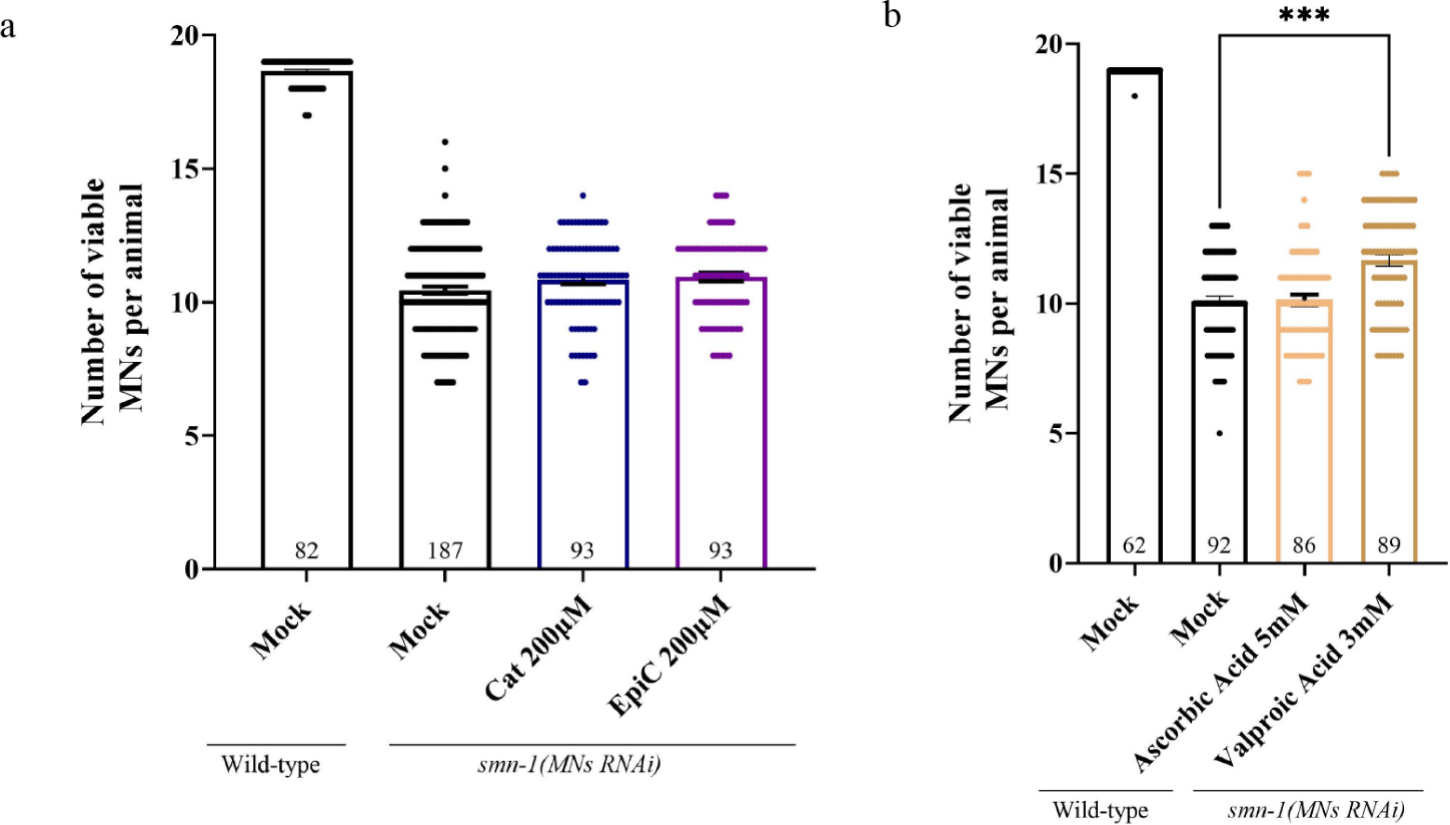
Catechins and ascorbic acid do not rescue the *smn-1* knockdown-induced early sign of neurodegeneration. a) Wild-type animals expressing the *oxIs12* transgene in mock conditions present an average of 18.7 visible (GFP-expressing) nondegenerated motoneurons (expected to be 19 per animal). *smn-1­(MNs RNAi)* animals show less visible GFP-expressing MNs (10.4) in the mock, a phenotype that is not rescued by catechins. Mock is DMSO 1%. b) Wild-type animals expressing the *oxIs12* transgene in mock conditions present an average of 18.9 visible (GFP-expressing) nondegenerated motoneurons (expected to be 19 per animal). Ascorbic acid 5 mM and valproic acid 3 mM were tested on SMA animals to score for visible (GFP-expressing) and only valproic acid rescued this phenotype; mock is water. Bars represent the mean of the number of visible motoneurons; each dot represents the number of visible MNs in a single animal; and n is the number of animals analyzed. The experiment was performed blindly as triplicate; SEM images are shown. Asterisks indicate a value significantly different (***p = 0.0002) when compared with solvent alone (mock), as calculated with One-Way ANOVA, Kruskal–Wallis nonparametric test, Dunn’s multiple comparison.

## 4 Discussion

Diet is among the most important factors impacting an individual’s health. The increasing worldwide population coupled with the evidence that the westernized diet, characterized by high fat, high sugar, high meat, and low plant fiber consumption, has been implicated as a risk factor for several diseases,^63,64^ calls for increasing efforts to exploit additional and sustainable sources of plant nutraceuticals for the long-term prevention and treatment of inflammation-related disorders, neurodegenerative disorders and major chronic diseases, including cancer. Polyphenols are among the phytochemicals with marked effects on the prevention of oxidative stress-associated pathologies. Within polyphenols, flavan-3-ols, both in their monomeric and polymerized forms (i.e., PAs), are known as potent antioxidant and anticancerogenic compounds.^6^ Yet, their biological activities depend on many factors, with composition and degree of polymerization being the most important ones. To explore additional, sustainable source of PAs with respect to the well-characterized ones (i.e., grapevine seeds, tea leaves) here we turned to the *Lotus* genus, which includes species with contrasting levels of PAs in their foliage. The goal was to gain preliminary evidence of the bioactivity of these compounds by comparing the acetone-soluble and insoluble fractions of closely related species on *in vitro* and *in vivo* models of neurodegenerative processes. Notably, the activity of these fractions has been compared with that of the pure chemicals that represent the main components of such as fractions.

### 4.1 *Lotus spp*. as an Alternative Source of PAs for Human Health

Several features prompted us to exploit *Lotus spp*. as sustainable sources of phytochemicals for human health beyond their traditional use as forage legumes. The potential high biomass production, the adaptability to environmental and climatic conditions that prevent the growth of the most valuable forage legumes for animal feeding such as alfalfa and soybean, coupled with the ameliorative function of *Lotus spp*. on soil fertility due to the symbiosis with nitrogen-fixing *Rhizobium spp*., are some of these features.^19^ Thus, along a wild and leaf PA-rich accession of *Lc* here we employed the leaf PA-depleted *Lt* and an interspecific *Lc* × *Lt* named *Lh2*. This phylogenetically related plant material has been already characterized from the molecular and metabolic point of view.^22–24^ As expected, the three genotypes when grown in the glasshouse to carry out the present study showed a marked difference in the content of PAs, for both the acetone-soluble and insoluble PA fractions. *Lt* leaves contained only traces of both fractions, the highest levels were in the wild parent *Lc*, while the hybrid showed intermediate levels of the soluble fraction and the acetone-insoluble fraction closer to that of the PA-rich parent than the PA-depleted parent. These differences among species were noted all over the growing season, corroborating the finding that they are genetically determined.^22^ The *Lc* acetone-soluble PA fractions mainly consist of the flavan-3-ol monomers and dimeric PAs, whereas the acetone-insoluble fraction consists of oligo- and polymeric-PAs. C and EC are known as the main terminal and extension units, respectively, in *L. corniculatus* PA. Notably, while the UPLC analysis of the depolymerized acetone-insoluble fraction showed that PA from *Lh2* mainly releases Cya those from the wild parental line, *Lc*, primarily releases delphinidin. Not only this observation corroborates what was initially observed by thin-layer chromatography assay on the same genotypes^22^ but it also might contribute to explain the difference in the biological activity of the acetone-insoluble fractions of *Lc* and *Lh2*.

#### 4.1.1 Acetone-Soluble and Insoluble Fractions of Leaves from Three *Lotus* Genotypes Show Differences on Neuroprotective Activities Depending on the Content of PAs

To test the hypothesis that the neuroprotective role of leaf extracts from genetically related *Lotus spp*. might depend on the content of PAs,^65^ we challenged SH-SY5Y cells and a *C. elegans* SMA model with both acetone-soluble and insoluble fractions from the parental lines, *Lc* and *Lt*, and their hybrid *Lh2*. Interestingly, the acetone-soluble fraction of *Lt* but not that from *Lc* and *Lh2* showed morbidity against SH-SY5Y cells. This difference in the efficacy of the extracts can be linked to the absence of flavan-3-ols and their oligomers in the *Lt* fraction. The addition of increasing doses of C to the acetone-soluble and acetone-insoluble fractions of *Lt* contributed in fact to increase cell viability, confirming this hypothesis. In line with the data on SH-SY5Y cells are those from the SMA model of *C. elegans*: only the acetone-soluble extracts for the PA-richest fraction (*Lc*) displayed a significant reduction in the number of dying MNs. The *Lh2* acetone-soluble extracts also showed a decrease, albeit not significant, in the number of dying MNs with respect to the control (mock).

The therapeutic effects of C and its oligomers against neurodegenerative diseases have been already reported by other studies with human and animal models. In fact, C can have anti-inflammatory effects by suppressing inflammatory pathways and cytokines as well as antioxidant effects such as chelating metal ions and scavenging radicals. C might also reduce the phosphorylation of tau proteins, the aggregation of amyloid-beta, and apoptotic proteins release while increasing dopamine levels [reviewed in ref 66].

Similar results were obtained with the butanol-HCl hydrolyzed acetone-insoluble fractions, as those from *Lh2* and *Lc* increased SH-SY5Y cell viability, although only at the highest dosage. Once again, these results get along with those obtained with the *C. elegans* SMA model, since the acetone-insoluble fractions of *Lc* and *Lh2* significantly reduced the number of dying MNs, with the fraction from the former species being more effective than the latter.

All in all, the present results show that differently from *Lc* and *Lh2* leaf extracts, those from *Lt* are toxic to SH-SY5Y cells and do not exert any positive effects on *C. elegans*. In the light of the competence of this species to accumulate several classes of flavonoids, flavonols *in primis*, this was indeed unexpected.^67–69^ Our data demonstrate that the toxicity of acetone-soluble and insoluble fractions of *Lt* can be halted or attenuated by pure C and that the acetone-insoluble extracts from either *Lh2* and *Lh* halt or attenuate the toxicity of *Lt* acetone-insoluble fraction.

### 4.2 Flavan-3-ols and Cya but Not Que Prevent Neurodegenerative Process

A large body of evidence confirms the neuroprotective roles exerted by flavonoids.^70–72^ Since the *Lotus* genotypes studied here were selected for their differences in PAs, both regarding the acetone-soluble PA fractions, mainly consisting of C and EC, and the acetone-insoluble fractions, which release anthocyanins upon acidic hydrolysis, we decided to test the activity of the pure compounds C, EC, Cya, and the flavonol Que, as a control, on the same *in vitro* and *in vivo* neurodegenerative models employed to test the plant extracts. Here we show that both flavan-3-ols and Cya play a protective role in the human cell line SH-SY5Y model for neurodegenerative studies. Not only were these compounds in a range of 50 −150 μM not harmful to these cells, but at their highest dosage (200 μM) they further increased cell viability. In contrast, the flavonol Que had no impact on cell viability at any dosage tested. These findings highlight a clear difference among flavonoids in their effectiveness in our cell model. This difference was also confirmed when the same cell line was challenged with the neurotoxic agent 6-OHDA. The viability of cells grown in the presence of a toxic (50 μM) level of 6-OHDA, increased significantly in the presence of C, EC, and Cya in the 100–200 μM range. The same did not occur with Que at any dosage tested.

When we turned to the *in vivo* model for neurodegenerative studies, that is the *C. elegans* SMA model, we obtained an even finer functional distinction among the different classes of flavonoids. Both C and EC reduced the number of dying MNs when tested at both 200 and 400 μM, with the last dosage proving even more effective than 200 μM. Conversely, Cya exerted a protective role only at the lower dosage, while Que did not preserve MNs from death either at 200 or at 400 μM. Since Cya, but also valproic acid, showed a rescue at a precise concentration, which was not the highest, this strongly suggests a bell-shaped dose–response curve that should be further analyzed by testing more doses. This type of curve is not unexpected since many studies have recognized the occurrence of nonmonotonic dose–response curves in organisms’ responses to nutrients.

Thus, the results from both models convey to show that flavan-3-ols and Cy, much more than the flavonol Que, play a critical protective role on cell and animal viability. We cannot exclude that Que may exert a protective role on our models at different dosages than those tested here; however, we note that flavonols could indeed pose a threat to cells. Indeed, Que was efficacious in other *C. elegans* models. For example, 100 μM Que was efficient in preventing amyloid-beta-induced detrimental effects^73^ *in vitro* and in a *C. elegans* model for Alzheimer’s disease, and it is tempting to speculate that the different effect can be explained by a disease-specific activity of Que, more than to a difference in concentration. Another important issue to take into account is the poor solubility and low mucosal permeability of Que, which can cause a lower bioavailability *in vivo*. In fact, Que’s neuroprotective effects on a *C. elegans* model of Parkinson’s disease were highly increased when a quercetin-loaded nanoemulsion was used.^74^ In this respect, testing lower doses of the individual molecules would be interesting to better understand the physiological window of efficacy.

A diet rich in Que, which is ubiquitously present in various vegetables, fruits, seeds, nuts, tea, and red wine,^75,76^ reduces the risk for oxidative stress-related chronic diseases such as diabetes, coronary heart disease, and stroke.^77,78^ However, antioxidants that scavenge free radicals might form products that absorb some of the reactivity of the scavenged radicals, a phenomenon known as the “quercetin paradox”.^79^ While offering protection against H_2_O_2_-induced DNA damage, Que in fact can be converted into a potentially toxic product. Thus, a higher bioavailability of flavonols in *Lt* than in *Lh2* and *Lc* acetone-soluble extracts could be evoked to explain the *Lt* toxicity to SH-SY5Y cells and their ineffectiveness in decreasing the number of dying motoneurons in the *C. elegans* SMA model.

Pure C and aqueous green tea extracts are known to increase lifespan and induce resistance to oxidative stress in *C. elegans*.^80–82^ For a broader comprehension of the effects of C on this animal model, we used two *smn-1 (MNs RNAi)* strains. The former allows to detect the accumulation of apoptosis-related fluorescence, a late event in neuronal death, but it does not allow to assess whether C might prevent MN degeneration at an early stage. To address this point, we challenged the *smn-1 (MNs RNAi); pMNs:GFP* strain with either C, ascorbic acid, selected as a control for antioxidant activity,^83^ or valproic acid, known to rescue MN degeneration at an early stage.^45^ While valproic acid increased the viability of GFP-expressing MNs, C, EC, and ascorbic acid did not. Therefore, C, EC, Cya, ascorbic acid, and valproic acid^45^ are all able to counteract apoptotic death, a late event observed in our SMA *C. elegans* model, while only valproic acid can prevent MN degeneration at earlier stages of degeneration. Thus, we argue that unlike valproic acid, pure C, EC, and flavan-3-ols present in the acetone-soluble *Lotus* fractions primarily rescue apoptotic death that occurs at late stages after the activation of the cell death pathway genes, possibly by virtue of their antioxidant activity, as ascorbic acid does. This result is confirmed by previous studies that showed the ability of C to efficiently block apoptosis through direct inhibition of caspases,^64^ which would explain why catechin and epicatechin, and possibly some of the natural extracts, are able to protect against neuronal death in our SMA model, without any effect on the prevention of early neurodegeneration, as demonstrated by the absence of any increase in motor neuron survival.

When considering the physiological relevance of the concentrations applied in our study, it is challenging to directly link them to *in vivo* exposures in higher animal models, as this depends on factors such as dietary intake, supplementation, and formulation. Evidence from randomized controlled or prospective trials that evaluate flavonoid supplementation with clinical outcomes, such as morbidity or mortality in humans, remains very limited. To bridge this gap, a pharmacokinetic framework will be essential to clarify how the tested concentrations relate to achievable exposure levels under real-life conditions.

In conclusion, while adding further evidence in support of the contention that C, EC, and Cya reduce mortality in SH-SY5Y cells,^84,85^ here we provide for the first time evidence that C, EC, and Cya but not Que play a neuroprotective role on a *C. elegans* model for SMA. Moreover, here we showed that acetone-soluble and insoluble fractions from PA-rich *Lc* are not cytotoxic, rather these fractions can increase viability in SH-SY5Y cells and decrease MN degeneration in *C. elegans* SMA model. Thus, a clear neuroprotective role for leaf extracts of *Lc* has been demonstrated by adopting both *in vitro* and *in vivo* models. Since oligomeric PAs are depolymerized to monomeric units during their transit along the intestinal tract of rats,^18^ it is conceivable that not only the acetone-soluble but also the acetone-insoluble fractions of PA-rich *Lotus spp*. might represent novel and sustainable sources of health-promoting phytochemicals. However, studies will be needed to assess whether and to what extent these *Lotus* fractions react with metabolites and microbiota present in the host’s digestive tract. Finally, our results pave the way for studies aimed at assessing whether the protective and antioxidant activities shown by *Lc* extracts are also shared with other PA-rich species belonging to the same Fabaceae family.

## Supplementary Material



## Notes

Supplementary Figure S1. UPLC-DAD profiles of acetone-insoluble *Lotus* extracts recorded at 520 nm. Supplementary Figure S2. Effects of the addition of increasing doses of C on the viability of SH-SY5Y cells treated with acetone-soluble (A) and acetone-insoluble (B)*Lt* fractions. Supplementary Figure S3. Effects of the addition of acetone-soluble and acetone-insoluble extracts from PA-rich *Lotus spp*. on the viability of SH-SY5Y cells treated with acetone-soluble (A) and acetone-insoluble (B)*Lt* fractions (PDF)

## Abbreviations

6-OHDA: 2,4,5-trihydroxyphenethylamine
ACNs: anthocyanins
C: (+)-catechin
Cya: cyanidin
DMSO: dimethyl sulfoxide
EC: (−)-epicatechin
EGC: (−)-epigallocatechin
ECG: (−)-epicatechin-3-gallate
EGCG: (−)-epigallocatechin-3-gallate
FW: fresh weight
PAs: proanthocyanidins
Que: quercetin
*Lc*: *Lotus corniculatus*
*Lt*: *Lotus tenuis*
*Lh2*: *L. corniculatus* × *L. tenuis* hybrid
SMA: Spinal Muscular Atrophy
