# Supplementary material for: Different Neuroprotective Activities of Proanthocyanidin-Enriched Fractions of Lotus Species
Source: ACS Omega. 2025 Dec 11;10(50):61480–93. doi: 10.1021/acsomega.5c06714 (PMC12750242; doi:10.1021/acsomega.5c06714)
Supplement: Supplementary file 1 [file ao5c06714_si_001.pdf]

SUPPORTING INFORMATION

Different neuroprotective activities of proanthocyanidin - enriched fractions of *Lotus* species

Authors

Maria Rachele Ceccarini[1], Nadia Mazzarella[2], Serena Visone[2], Pamela Santonicola[2], Antonella Camera[2], Federica Cieri[2,3], Federica La Rocca[2,4], Ilenia Matino[2], Giuseppina Zampi[2], Maria Cristina Valeri[5], Francesco Damiani[5], Francisco Jose Escaray[6], Oscar Adolfo Ruiz[6], Stefan Martens[7], Tommaso Beccari[1], Elia Di Schiavi[2*], Francesco Paolocci[5*]

*Co-corresponding authors

Affiliations

[1] Department of Pharmaceutical Science, University of Perugia, 06122, Perugia, Italy
[2] Institute of Biosciences and Bioresources, IBBR Naples division, CNR, Via P. Castellino 111, 80131, Napoli, Italy
[3] Department of Biology, University of Naples "Federico II", Napoli, Italy
[4] Department of Environmental, Biological and Pharmaceutical Sciences and Technologies, University of Campania "L. Vanvitelli", Caserta, Italy
[5] Institute of Biosciences and Bioresources, IBBR Perugia division, CNR, Via Madonna Alta 130, 06128 Perugia, Italy
[6] Chascomús Technological Institute (INTECH), School of Nanotechnology and Biotechnology (UNSAM-CONICET), Buenos Aires province, Argentina
[7] Fondazione Edmund Mach, Centro Ricerca e Innovazione, Via E. Mach, 1, 38098 San Michele all' Adige, TN, Italy

Short title: Tannins protect neurons from degeneration

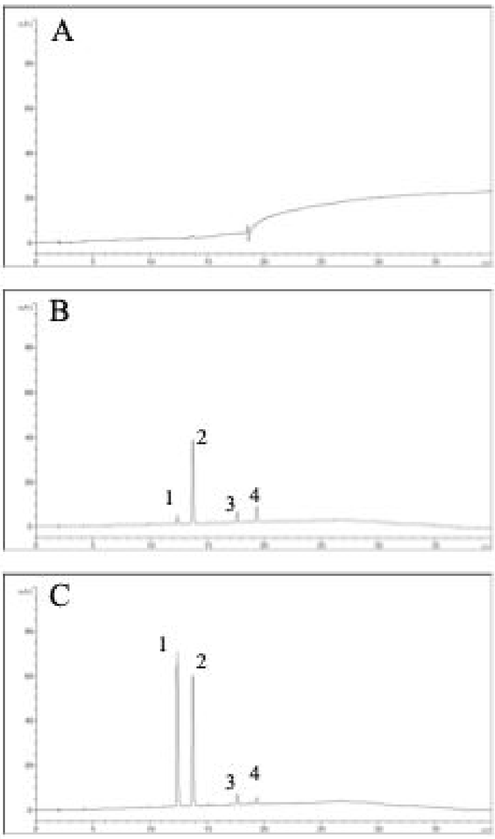

Legend: 1 – delphinidin; 2 – cyanidin; 3 – unknown anthocyanin 1; 4 – unknown anthocyanin 2

Supplementary Figure 1. UPLC-DAD profiles of acetone-insoluble *Lotus* extracts recorded at 520 nm. A: *Lt*; B: *Lh2*; C*: Lc.*

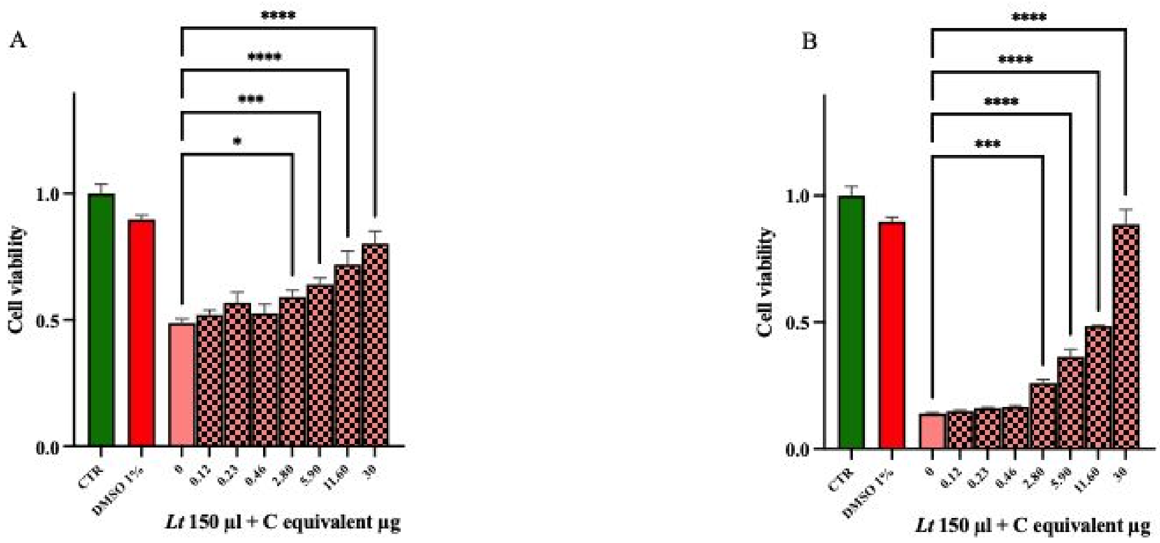

Supplementary Figure 2. Effects of the addition of increasing doses of C on the viability of SH-SY5Y cells treated with acetone-soluble (A) and acetone-insoluble (B) *Lt* fractions. Negative control (in green), positive control (in red), 150 µl of *Lt* extract (in pink), 150 µl of *Lt* extract plus C from 0.12 to 30 µg (squares in pink) are reported. Asterisks indicate a value significantly different (*$p<0.05$; ***$p<0.001$; ****$p<0.0001$) from animals treated with *Lt* alone (0 µg of C) as calculated with One Way ANOVA, Kruskal Wallis nonparametric test, Dunn's multiple comparison.

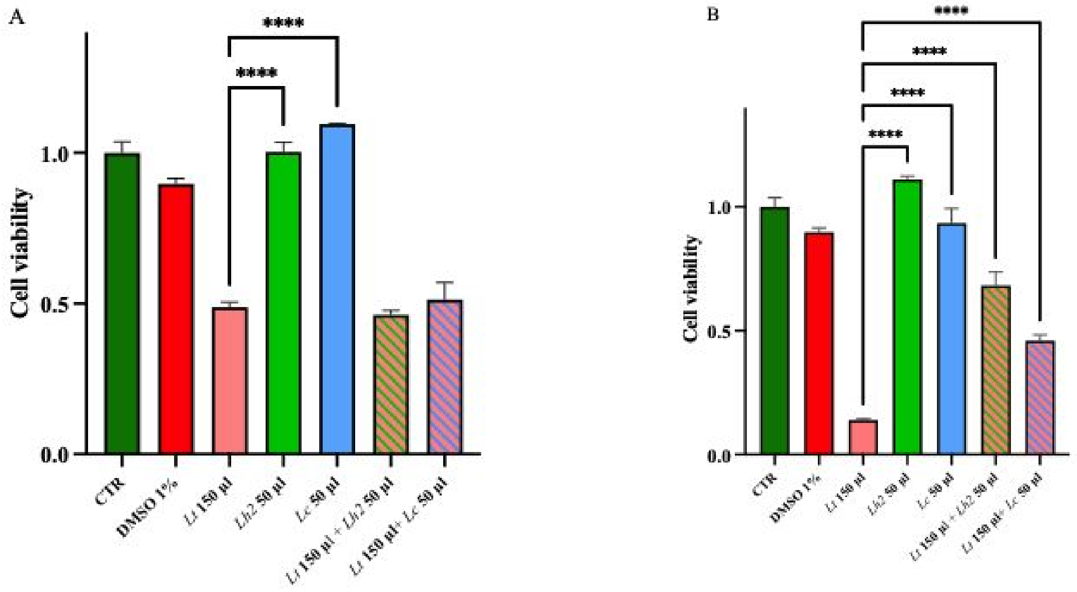

Supplementary Figure 3. Effects of the addition of acetone-soluble and acetone-insoluble extracts from PA-rich *Lotus* spp. on the viability of SH-SY5Y cells treated with acetone-soluble (A) and acetone-insoluble (B) *Lt* fractions. Negative control (in green), positive control (in red), 150 µl of *Lt* extract (in pink), 50 µl of *Lh2* extract (in light green), 150µl of *Lt* extract plus 50 µl of *Lh2* extract (in green and pink), 150 µl of *Lt* extract plus 50 µl of *Lc* extract (in pink and blue) are reported. Asterisks indicate a value significantly different (****p<0.0001) from animals treated with *Lt* alone (pink bar) as calculated with One Way ANOVA, Kruskal Wallis nonparametric test, Dunn's multiple comparison.
